# Photonic crystal L3 cavity laser fabricated using maskless digital photolithography

**DOI:** 10.1515/nanoph-2022-0021

**Published:** 2022-04-11

**Authors:** Minsu Kang, Heesoo Jin, Heonsu Jeon

**Affiliations:** Department of Physics and Astronomy, Seoul National University, Seoul 08826, Republic of Korea; Inter-university Semiconductor Research Centre, Seoul National University, Seoul 08826, Republic of Korea; Institute of Applied Physics, Seoul National University, Seoul 08826, Republic of Korea

**Keywords:** cavity lasers, light diffraction, maskless digital photolithography, photonic crystals, submicron patterns

## Abstract

Projection photolithography using an extreme-ultraviolet light source is the core technology that has enabled patterning on the scale of a few nanometers that is required for modern electronic chips. However, this high-end system is neither affordable nor needed for photonics where critical feature sizes are of 100s of nanometers (or of submicron). Although electron-beam lithography can provide a means for photonic device fabrication, it suffers from extremely low throughput. Therefore, a lithographic technique for submicron pattern generation at high throughput and low cost is in high demand. This group recently showed that maskless digital photolithography (MDPL), a convenient and versatile photolithographic technique that requires no photomask, could potentially address this demand by demonstrating photonic crystal (PhC) patterns with submicron periodicity and associated PhC band-edge lasers. In this paper, we report the fabrication of a PhC L3 cavity laser, which contains irregular air holes in terms of their positions and sizes, using the MDPL technique. Successful generation of such an aperiodic and nontrivial submicron pattern requires thorough understanding and scrupulous manipulation on light diffraction. Our achievements should provide the concrete foundation upon which compact, versatile, convenient, speedy, and economical lithographic tools for arbitrary submicron pattern generation can be developed.

## 1 Introduction

Nanometer-scale pattern generation can be easily achieved using several nanolithographic techniques, such as nanophotolithography [1], [2], [3], [4], [5], electron-beam lithography (EBL) [6, 7], nanoimprint [8, 9], scanning probe lithography [10], [11], [12], [13], and plasmonic lithography [14, 15]. Most notably, transistor fin pitch as small as 27 nm has been obtained by the projection photolithographic system equipped with an extreme-ultraviolet light source [5]. However, owing their extreme cost, low throughput, and insufficient stability, the use of these techniques is limited to certain applications or environments. For example, extreme-ultraviolet photolithography is affordable only for a few gigantic electronic chip manufacturers. Meanwhile, EBL suffers from extremely low throughput and small field size, although it has become the predominant choice for nanometer-scale patterning in research, development, and even small-volume commercial production. Under these circumstances, a compromised lithographic technique, characterised by a somewhat relaxed resolution but high throughput and low cost, is highly desirable. If available, this type of lithographic system would strongly appeal to certain applications and sectors, such as photonics and the low-end electronic industry where critical dimensions required are in the order of 100 nm (or submicron) or larger. We believe that *maskless digital photolithography* (MDPL) is a strong candidate to meet this requirement.

**Figure 1:**
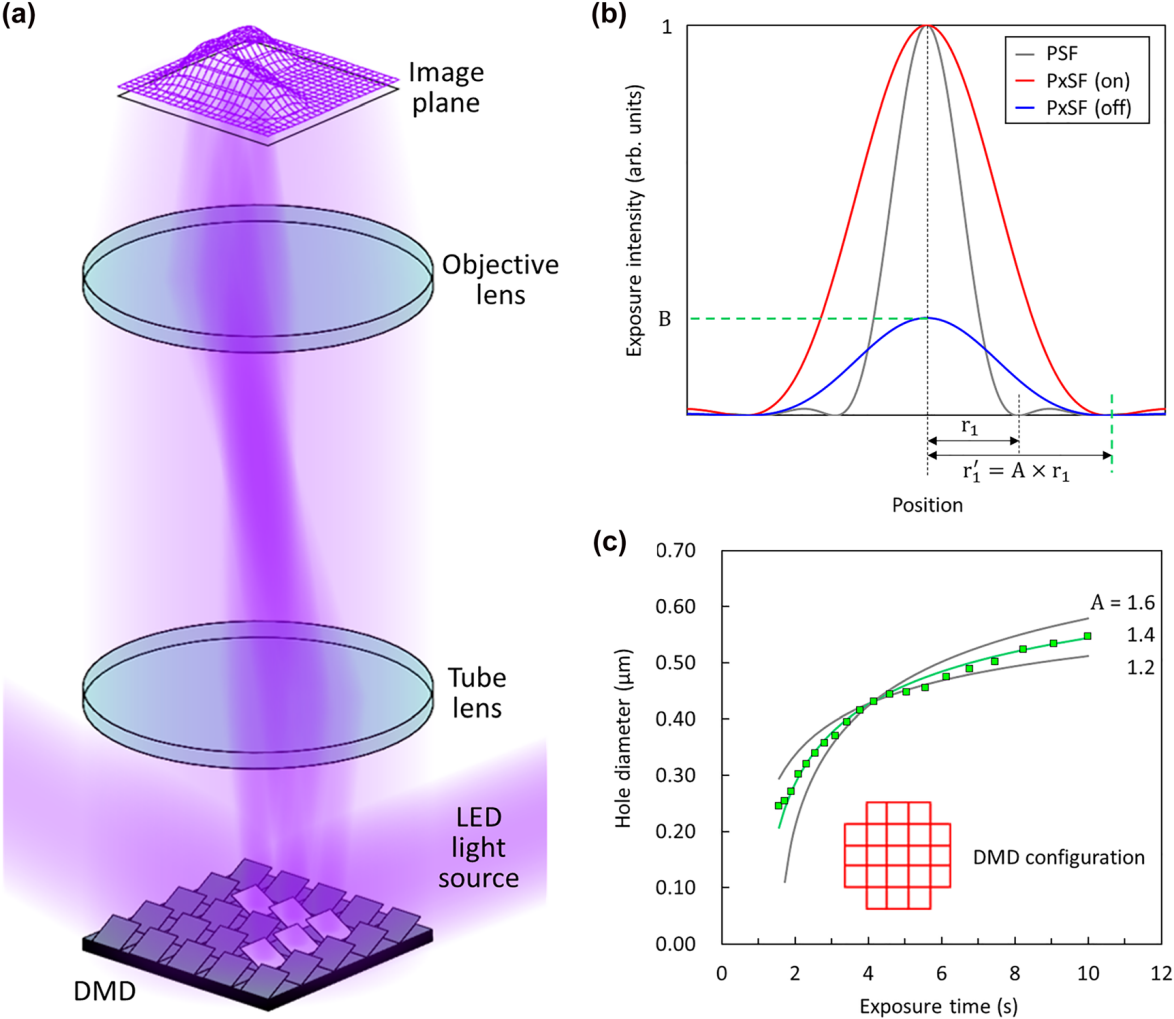
Light diffraction by DMD pixels. (a) Schematic of the submicron pattern formation on the image plane by diffracted lights from individual DMD pixels. (b) Diffraction profiles by single ‘on’ and ‘off’ DMD pixels (PxSFs) compared with that by a point source (PSF). *A* (>1) is the swelling factor of the PxSF from PSF, while *B* (<1) is the diffraction intensity of an ‘off’ pixel with respect to that of an ‘on’ pixel. (c) Experimentally determined air-hole pattern diameter (squares) as a function of exposure time, when a single air-hole pattern is generated using the group of 21 ‘on’ pixels shown in the inset. The solid curves are the calculation results for different *A* values.

MDPL is a micro photolithographic technique that is convenient and versatile as its pattern layouts can be configured dynamically and without photomask [16]. Further, it is affordable as it utilizes a combination of cheap light source, such as discharge lamps [17], [18], [19], [20], [21], [22], [23], solid-state lasers [24, 25], or light-emitting diodes (LEDs) [26], [27], [28], and electrically-addressable spatial light modulator chip known as digital-micromirror-device (DMD) [16], [17], [18], [19], [20], [21], [22], [23], [24], [25], [26], [27], [28] (Figure 1a). MDPL has rapidly replaced the existing contact/proximity-based micro photolithographic equipment that requires photomasks. Nonetheless, no systematic attempt has been made thus far to shrink the MDPL pattern size below 1 μm, except for a few proofs-of-concept [24, 25, 27]. Recently, this group demonstrated the first operating nanophotonic devices using the MDPL technique [28]. Specifically, we constructed a custom-designed MDPL system using a DMD chip with the smallest pixel pitch available (*p* = 5.4 μm) and a 200× objective lens (NA = 0.9), and then we fabricated photonic crystal (PhC) band-edge lasers composed of a two-dimensional (2D) submicron air-hole array. Although successful, the band-edge laser patterns were generated by trials and errors, which was possible because the corresponding structures are periodic and the constituent features are larger than the diffraction limit (∼360 nm; see Supplement of Reference [28]). When *arbitrary* submicron patterns are to be generated, however, such a smattering approach cannot work. Rather, it requires a careful bottom-up approach based on complete understandings and proper manipulations on exposure details by individual DMD pixels.

The present study identifies *diffraction* as the key to the successful generation of arbitrary submicron patterns by the MDPL as the spatial extent of diffracted light is of the same order as the feature size to be defined, and also addresses the question of how the fidelity between intended and actual patterns can be improved. As an intermediate milestone en route to the ultimate goal of completely arbitrary submicron patterns, we chose a PhC L3 cavity structure and demonstrated lasing action by implementing it on an InP-based multiple-quantum-well (MQW) epistructure. The PhC L3 cavity, known as the most advanced PhC cavity structure with the highest Q-factor [29, 30], includes not only missing air holes but also some air holes smaller than others. Thus, the air holes have neither periodic arrangement nor identical size, therefore structurally far more arbitrary than the previously demonstrated PhC band-edge laser structures [28]. Our results should be an important step toward the development of viable submicron photolithographic equipment with the aims of convenience (masklessness), speed (high throughput), and economy (simplicity and low cost). In particular, the writing speed is the utmost figure-of-merit that makes our MDPL stand out over EBL; our MDPL can generate typical PhC patterns 10–100 times faster than EBL, while requiring no sophisticated hardware for high vacuum or electron beam.

## 2 Diffraction by digital-micromirror-device pixels

The present study begins with the notion that diffraction must be considered and carefully manipulated if arbitrary submicron patterns are to be generated with good fidelity by MDPL. As feature sizes approach the wavelength of the exposure light, diffraction effects are unavoidable. It should be noted that our previous MDPL work on PhC band-edge lasers dealt with periodic patterns only so that the semi-empirical approach allowed us to bypass the diffraction effects [28]. Light diffraction by individual DMD pixels mainly triggers the following two effects: (1) spatial swelling of the image of each DMD pixel and (2) nonzero (or finite) contribution by ‘off’ DMD pixels. The overall exposure profile due to a specific DMD pixel configuration can then be obtained by superposing all the diffraction profiles of the individual DMD pixels, including both ‘on’ pixels and ‘off’ pixels (Figure 1a).

The spatial distribution of the diffracted light from a single DMD pixel can be modelled by the pixel spread function (PxSF) [23, 31], [32], [33], [34], modified from the point spread function (PSF) to handle an extended diffraction source. In the PxSF model, the diffraction pattern of a single DMD pixel is assumed to be still given by the Airy function, but effectively swollen by a factor of A. Thus, the first diffraction minimum occurs at $r_{1}^{\prime }=A\times r_{1}\left(A>1\right)$ , instead of $r_{1}\approx 0.61\frac{\lambda }{NA}$ , where *λ* and NA are the wavelength of the exposure light and the numerical aperture of the imaging system, respectively. The PxSFs for single ‘on’ and ‘off’ DMD pixels are schematically illustrated in Figure 1b, along with the PSF for comparison. To quantitatively assess the contribution of an ‘off’ pixel, we directly measured and compared the light intensities recorded by a photodetector located at the image plane when the entire DMD chip was switched ‘on’ and ‘off’. The intensity ratio between them was measured to be $B=I_{0}^{\text{off}}/I_{0}^{\text{on}}\approx 1/206$ . Although the ‘off’ pixel contribution seems insignificant, it is not ignorable when a pattern to be defined includes many ‘off’ pixels, PhCs being a good example. Then, we defined a series of circular air-hole patterns for various exposure times and measured the resultant hole diameters to determine the swelling factor A for our MDPL system; note that the circular air-hole is the basic element that composes PhC patterns. During the experiment, the air-hole pattern was defined by the DMD configuration shown in the inset of Figure 1c, composed of 21 ‘on’ DMD pixels. When fitting the measured data using a theoretical prediction with the ‘off’ pixel effect (or the *B* coefficient) is considered, we conclude that *A* = 1.4 provides the best fit, as shown in Figure 1c. We are now fully informed of the diffraction characteristics of our MDPL system.

## 3 Exposure conditions for photonic crystal patterning

**Figure 2:**
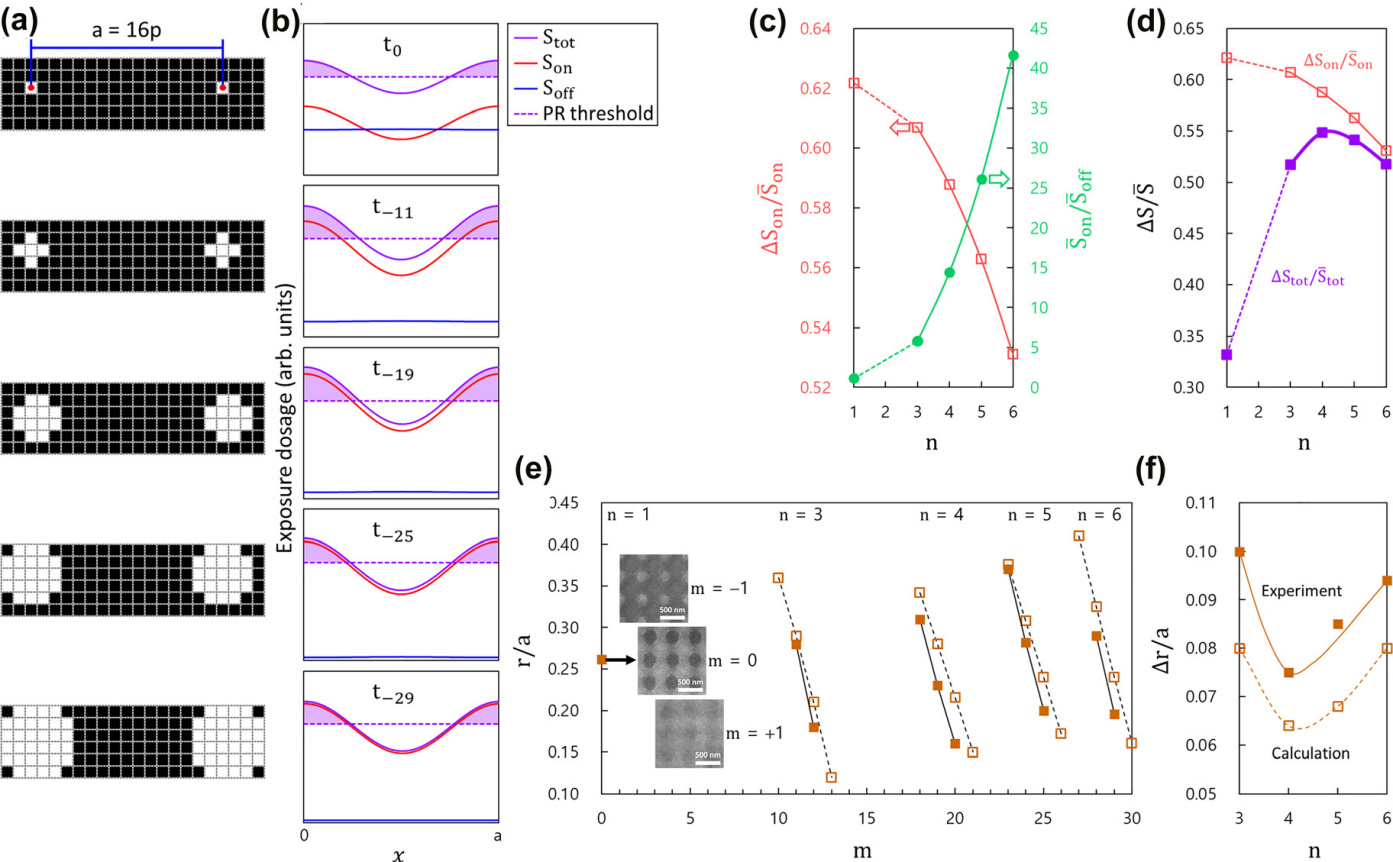
Exposure conditions for PhC backbone generation. (a) Exposure maps for the generation of square lattice PhC, each air-hole pattern defined by *n* × *n* (−4) DMD pixels: (from top) *n* = 1, 3, 4, 5, and 6. Only one lattice period is shown in the horizontal direction. (b) Exposure dosage profiles calculated for the exposure maps illustrated in (a). Each figure shows the profiles produced by the ‘on’ pixels only (*S*_on_, red), the ‘off’ pixels only (*S*_off_, blue), and both the ‘on’ and ‘off’ pixels (*S*_tot_, violet), while the dotted line represents the PR threshold. Refer to the manuscript for the exposure time *t*_−m_. (c) Average exposure dosage ratio between the ‘on’ and ‘off’ pixels ( ${\bar{S}}_{\text{on}}/{\bar{S}}_{\text{off}}$ , solid circles) and normalized amplitude of the exposure dosage profile by the ‘on’ pixels ( $\Delta S_{\text{on}}/{\bar{S}}_{\text{on}}$ , open squares), both as a function of *n*. (d) Normalized total exposure dosage amplitude ( $\Delta S_{\text{tot}}/{\bar{S}}_{\text{tot}}$ , solid squares) as a function of *n*. Although redundant, $\Delta S_{\text{on}}/{\bar{S}}_{\text{on}}$ is shown again to emphasize that it approaches to $\Delta S_{\text{tot}}/{\bar{S}}_{\text{tot}}$ as *n* becomes large. (e) Relationship between the air-hole size (*r*/*a*) and *m*, plotted for different *n*’s. The solid squares are from the experiments, while the open squares are deduced from the calculation results. The insets are SEM images of the test samples generated for *n* = 1 at three different exposure times: *m* = −1, 0, and +1. (f) Air-hole size change per exposure time step (Δ*m* = 1) as a function of *n*. All the data are deduced from (e).

The next step is finding the optimum exposure conditions for patterning our PhC backbone structures that are composed of a periodic air-hole array while fully considering the diffraction effects. As shown in Figure 2a, a circular air-hole pattern is defined by (*n* × *n* − 4) DMD pixels, where *n* = 3, 4, 5, and 6; four pixels are removed to round off the otherwise sharp corners. A square lattice PhC with lattice constant *a* = 16*p* (≈455 nm) was selected as the model structure that represents the typical PhC structures. Figure 2b shows the cross-sectional exposure dosage profiles calculated for the DMD configurations shown in Figure 2a. Here, *S*_on_ is by the ‘on’ pixels only, *S*_off_ by the ‘off’ pixels only, and *S*_tot_ by both the ‘on’ and ‘off’ pixels. They were calculated using the experimentally determined diffraction coefficients: *A* and *B*. The dotted line in each figure represents a suggested photoresist (PR) threshold so that the PR exposed above the threshold (shaded in the figure) is to be developed. We assumed that the exposure time was adjusted by a geometric progression with a common ratio of 1.1. The proper exposure time, *t*_−*m*_ = *t*_0_(1.1)^−*m*^, should then be the one that makes the PR threshold align close to the median of the total exposure profile, where *t*_0_ is the proper exposure time for *n* = 1. The results are *t*_−11_ (≈0.35*t*_0_), *t*_−19_ (≈0.16*t*_0_), *t*_−25_ (≈0.09*t*_0_), and *t*_−29_ (≈0.06*t*_0_) for *n* = 3, 4, 5, and 6, respectively.

To evaluate the quality of the exposure profiles, the ratio of the average exposure dosages by the ‘on’ and ‘off’ pixels $\left({\bar{S}}_{\text{on}}/{\bar{S}}_{\text{off}}=\left[S_{\text{on}}^{\text{max}}+S_{\text{on}}^{\text{min}}\right]/\left[S_{\text{off}}^{\text{max}}+S_{\text{off}}^{\text{min}}\right]\right)$ and the normalised amplitude of the exposure dosage profile by the ‘on’ pixels $\left(\Delta S_{\text{on}}/{\bar{S}}_{\text{on}}=2\left[S_{\text{on}}^{\text{max}}-S_{\text{on}}^{\text{min}}\right]/\left[S_{\text{on}}^{\text{max}}+S_{\text{on}}^{\text{min}}\right]\right)$ are deduced from Figure 2b and plotted in Figure 2c, both as a function of *n*. As *n* increases, ${\bar{S}}_{\text{on}}/{\bar{S}}_{\text{off}}$ increases monotonically, whereas $\Delta S_{\text{on}}/{\bar{S}}_{\text{on}}$ behaves in the opposite manner. While the former is evident, the monotonic decrease of $\Delta S_{\text{on}}/{\bar{S}}_{\text{on}}$ is because the individual air-hole pattern size increases in proportion to *n*, which effectively flattens out the *S*_on_ profile. From the photolithography viewpoint, the larger both ${\bar{S}}_{\text{on}}/{\bar{S}}_{\text{off}}$ and $\Delta S_{\text{on}}/{\bar{S}}_{\text{on}}$ , the better. This implies that the two factors compete with each other and the optimum exists somewhere in the middle. In fact, the normalised amplitude of the total exposure dosage profile $\left(\Delta S_{\text{tot}}/{\bar{S}}_{\text{tot}}=2\left[S_{\text{tot}}^{\text{max}}-S_{\text{tot}}^{\text{min}}\right]/\left[S_{\text{tot}}^{\text{max}}+S_{\text{tot}}^{\text{min}}\right]\right)$ , which represents the overall exposure characteristics, exhibits a maximum at *n* = 4, as shown in Figure 2d. Note that $\Delta S_{\text{tot}}/{\bar{S}}_{\text{tot}}$ gradually approaches $\Delta S_{\text{on}}/{\bar{S}}_{\text{on}}$ as *n* increases, implying that the contribution of the ‘on’ pixels becomes dominant for large values of *n*. We conclude that *n* = 4 is the best option for the generation of our PhC pattern.

To confirm the validity of the calculation results, we first experimentally determined *t*_0_ for *n* = 1, which is suitable for our MDPL system and experimental conditions. We found that *t*_0_ (*m* = 0) has a quite narrow window; hence, the exposure times adjusted by one step up (*m* = +1) or down (*m* = −1) from *t*_0_ resulted in a PR pattern either overdeveloped or underdeveloped, respectively, as shown in the inset photographs in Figure 2e. After *t*_0_ is identified, the proper exposure times for other *n* values can be estimated by the *m* values determined earlier. Figure 2e summarises the trend of air-hole pattern size versus *m* for different values of *n*. There is a great degree of agreement between the experiments and calculations. The average changes in the air-hole size (Δ*r*/*a*) per exposure step (Δ*m* = 1) are extracted from Figure 2e and plotted in Figure 2f. Except for a finite offset, both the experimental and calculated results behave similarly, exhibiting a minimum at *n* = 4. From a photolithographic point of view, the smaller response in the air-hole pattern size to the change of the *m* value, the more preferable. This ensures that *n* = 4 is indeed the optimum DMD configuration for the generation of our PhC patterns.

## 4 Simulations on patterning the photonic crystal L3 cavity

**Figure 3:**
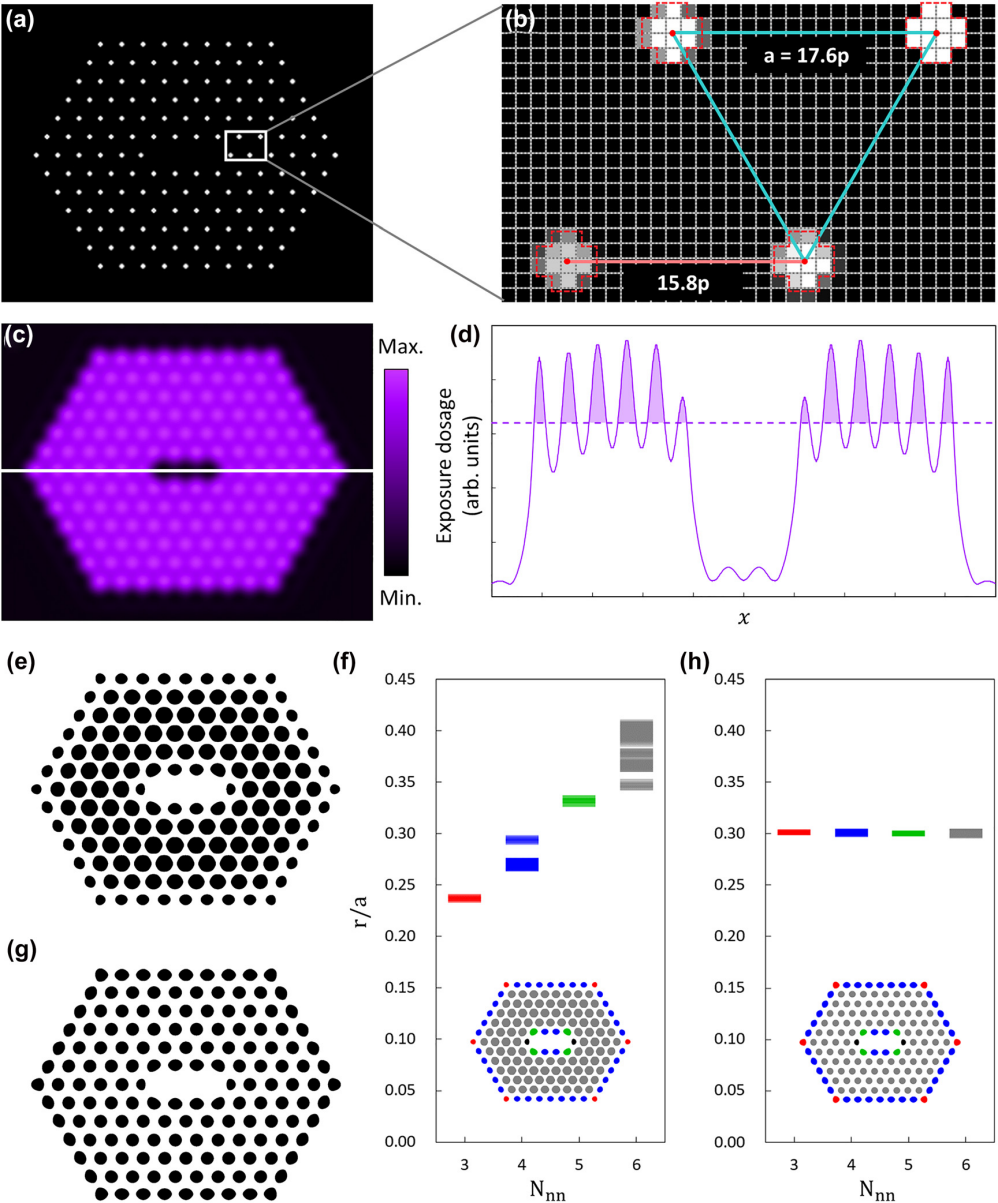
Simulations on PhC L3 cavity patterning. (a) Exposure map for the PhC L3 cavity pattern formation. (b) Magnified view of an area near the L3 cavity end. Each air-hole pattern is defined based on the grayscale exposure method, using the DMD pixel configuration of *n* = 4. (c) 2D exposure dosage profile on the image plane, calculated for the exposure map in (a). (d) Cross-sectional exposure dosage profile along the line intersecting the L3 cavity, denoted by (c). The dotted line indicates a desirable PR threshold. (e) Simulated PR pattern deduced from (c). (f) Air-hole pattern sizes (*r*/*a*) versus the number of the nearest neighbors (*N*_nn_) before the exposure corrections. The two air holes at the L3 cavity ends, which are intentionally designed smaller, are excluded. (g) Simulated PR pattern after the exposure corrections. (h) Relationship between *r*/*a* and *N*_nn_ after the exposure corrections. Note that the two air holes at the cavity ends are excluded. In the insets in (f) and (h), the air holes of different *N*_nn_ are distinguished in different colors.

To prove that accurate consideration of diffraction can indeed lead to successful submicron pattern generation with good fidelity, we chose the PhC L3 cavity structure as a testbed, which allegedly possesses the highest quality factor among PhC-based nanocavities [29, 30]. The PhC L3 cavity is formed by removing three air holes in a hexagonal lattice PhC backbone structure. Its Q-factor is then improved by reducing the size of the two air holes at the cavity ends and subsequently shifting their positions outward. Therefore, the air holes constituting the PhC L3 cavity are neither identical in size nor periodic in arrangement. Figure 3a shows the MDPL exposure map used to form the PhC L3 cavity structure. As shown in Figure 3b, each air-hole pattern was defined by the DMD array configurations of *n* = 4 with the application of the grayscale exposure method [28]. The PhC backbone has a hexagonal lattice structure with a lattice constant and air-hole radius of *a* = 17.6p ≈ 503 nm and *r*/*a* ≈ 0.40, respectively. Note that the air hole in the lower-left corner in Figure 3b, which terminates one of the two cavity ends, is smaller and shifted, as mentioned previously. Figure 3c shows the exposure dosage profile on the image plane, which was calculated from the exposure map in Figure 3a. The cross-sectional profile along the horizontal line marked in Figure 3c is plotted in Figure 3d. With an appropriate PR threshold – the dotted line in Figure 3d – a PR pattern that would emerge after development can be deduced, as shown in Figure 3e. The overall PR pattern appears to be reasonably close to the intended PhC L3 cavity structure, but not quite yet. In particular, the air-hole patterns immediately adjacent to the regions where there is no air-hole pattern – around the cavity and along the device edges – are significantly smaller and non-circular.

To address the issue of low fidelity in the PR pattern, a deeper consideration should be given to diffraction: the spatial extent of each air-hole pattern is wide enough to affect the formation of adjacent (or nearest neighbour) air-hole patterns. In fact, the air-hole pattern size is strongly correlated with the number of nearest-neighbour air holes *N*_nn_, as shown in Figure 3f, where the sizes of the air holes in Figure 3e are plotted as a function of *N*_nn_ (=3, 4, 5, and 6). Evidently, the higher the *N*_nn_, the larger is the air-hole size. To make all the air-hole patterns equal in size, the exposure times of the ‘on’ pixels must be adjusted appropriately for different *N*_nn_. Figure 3g shows the calculated PR pattern after the exposure corrections, from which Figure 3h is deduced. The air-hole patterns become uniform in size, and their size fluctuations are reduced. We note that the air holes along the boundaries of the cavity as well as the entire device remain noncircular due to the asymmetric exposure conditions. We expect that a more considerate attention to higher-order diffraction effects and grayscale exposure could bring about further improvement.

## 5 Photonic crystal L3 cavity laser

**Figure 4:**
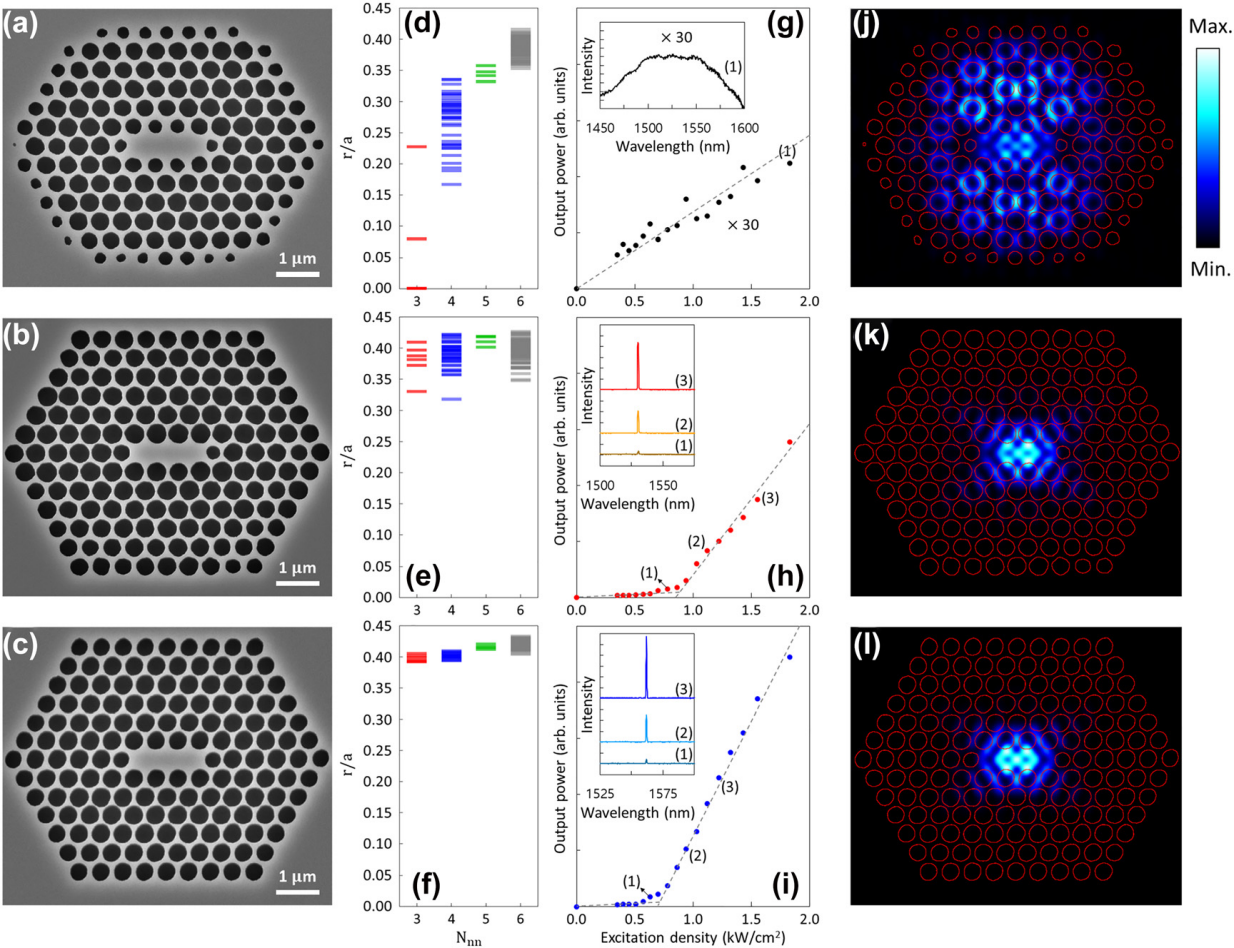
PhC L3 cavity lasers. (a–c) SEM images for the PhC L3 cavity laser devices, fabricated by the MDPL without (a) and with (b) exposure corrections for the nearest-neighbor effect and by EBL (c). (d–f) Air-hole sizes measured for the PhC L3 cavity laser devices, fabricated by the MDPL without (d) and with (e) exposure corrections and by EBL (f). The data are deduced from the corresponding SEM images in (a–c). (g–i) L–L curves measured for the PhC L3 cavity lasers, fabricated by the MDPL without (g) and with (h) exposure corrections and by EBL (i). The insets show emission spectra measured from the corresponding devices at the input power levels indicated. (j–l) Simulated modal profiles for the PhC L3 cavity laser devices, fabricated by the MDPL without (j) and with (k) exposure corrections and by EBL (l). Each device shows the cavity mode of the highest Q-factor.

Based on the design calculations, we fabricated the PhC L3 cavity laser devices in an air-bridge membrane form, using an InGaAsP MQW epistructure emitting in the 1550-nm communications wavelength range; the fabrication details are described in Methods. Figure 4a and b show the scanning electron microscope (SEM) images of the PhC L3 cavity laser devices fabricated before and after the corrections on the nearest-neighbor effect in the MDPL exposure, respectively; therefore, they correspond to Figure 3e and g. Evidently, the uniformity in the air-hole sizes was significantly improved after the exposure corrections. It should be noted that the two air holes at the cavity ends are intentionally reduced. Individual air-hole sizes were measured from the SEM images and plotted as a function of *N*_nn_ in Figure 4d and e, which are qualitatively consistent with Figure 3f and h, respectively. To directly assess the qualities of the PhC L3 cavities, both devices were optically excited using a 1064-nm laser diode in pulsed mode. The measured L–L relationships are plotted in Figure 4g and h. Only the device fabricated with exposure corrections lased with a clear threshold. Emission spectra measured at a few representative excitation levels also exhibit significant contrast (as shown in the insets: broad spontaneous emission versus sharp stimulated emission in single mode).

For comparison, we also fabricated a nominally identical PhC L3 cavity laser device using an EBL. Shown in Figure 4c, f, and i are its SEM image, air-hole size variations, and L–L curve with emission spectra. As shown in Figure 4f, the variations in the air-hole sizes are substantially smaller than those fabricated by the MDPL, which is simply due to the intrinsically much smaller diffraction for electrons. Consequently, the lasing threshold power density (∼680 W/cm^2^) was lower than that of the MDPL device (∼840 W/cm^2^). These observations are consistent with the finite-difference time-domain (FDTD) simulation results for the cavity structures digitised from the SEM images; the highest modal Q-factors are 8.6 × 10^2^, 1.71 × 10^4^, and 1.95 × 10^4^ for the cavities shown in Figure 4a, b and c, respectively. Figure 4j, k, and l show the corresponding cavity modes of the highest Q-factors and are, thus, presumed to be the lasing modes. The cavity modes are characterised by extended and partially leaky, slightly asymmetric, and highly symmetric modal profiles. Although much work needs be done, we have proven that sophisticated nanophotonic devices with good structural quality and performance characteristics can be fabricated by MDPL.

## 6 Discussion

To prove that the MDPL is capable and versatile enough to generate arbitrary submicron patterns, we have scrutinised light diffraction by individual DMD micromirrors, including both ‘on’ and ‘off’ pixels. The resultant diffraction effects were considered when establishing exposure maps based on the grayscale exposure scheme, which is necessary to overcome the digital nature of the DMD chip. A 2D PhC L3 cavity structure was selected as a testbed because it is composed of submicron air holes that are neither periodic nor equal in size; thus, it is arbitrary. Diffraction from each DMD pixel was found to be wide enough to affect the formation of the nearest-neighbor air-hole patterns. The exposure map for the PhC L3 cavity was revised to reflect the nearest-neighbor effect. The PhC L3 cavity structure implemented in an InGaAsP MQW epilayer exhibited a single-mode lasing action, and its performance was comparable to that of the reference device generated using EBL. The present study should stimulate the development of low-cost and high-throughput lithographic equipment for arbitrary submicron pattern generation. By providing more accurate and proper considerations of diffraction effects (e.g. with the aid of machine learning and deep learning), we strongly believe that this goal can be attained in the near future. In parallel to our efforts, the photolithographic resolution could be improved by upgrading the system hardware, such as, by adopting *immersion lithography* to increase the NA of the system [35] or by employing an exposure light source emitting at a much shorter wavelength. A larger DMD chip is also highly demanded as it could enlarge the write-field size and thus enhance the lithographic throughput.

## 7 Methods

### 7.1 Device fabrication

A 50-nm-thick silicon nitride hard mask layer was deposited on an InGaAsP MQW epistructure by plasma-enhanced chemical vapor deposition (310PC, Surface Technology Systems) at a process temperature of 300 °C. A positive PR (AZ MiR 701, Merck) was thinned and spin-coated onto the hard mask layer to a nominal thickness of 190 nm. The PhC patterns were then written using a customised MDPL system. After development in a PR developer (AZ 300MIF, Merck), the patterns were transferred sequentially to the hard mask layer and the MQW layer using reactive ion etching (RIE 80 Plus, Oxford Instrument). The PR and hard mask layers were then removed by dry etching. Finally, the underlying InP sacrificial layer was wet-etched through air holes in a diluted HCl solution to complete the air-bridge device fabrication.

### 7.2 Microphotoluminescence measurements

The emission spectra from the PhC L3 cavity laser devices were recorded using a home-made fiber-based microphotoluminescence setup [36], in which a cleaved butt-end fiber probe tip with a 62.5-μm core diameter was used for simultaneous optical excitation and collection. The fiber tip was brought in close proximity to the device surface, while the other end was fused to a 1 × 2 fiber coupler. The input port of the coupler was connected to a 1064-nm pulsed laser diode (PSL10, Multiwave Photonics), operating at a 500-kHz repetition rate with a 20-ns pulse duration, while the output port was connected to an optical spectrum analyser (MS9740A, Anritsu) for spectral analysis.

### 7.3 Cavity mode simulations

Numerical simulations based on the FDTD method were performed using a commercial FDTD software package (FDTD Solutions, Lumerical Solutions). For each model structure, the simulation domain size was 12 × 10 × 3 μm, and the spatial resolution was set to 100 pixels/μm. We employed the full 3D FDTD method with a refractive index *n* = 3.4 for the InGaAsP PhC waveguide slab. The model PhC cavity structures used in the simulations were identical to the real devices examined experimentally because the SEM images were digitised to construct the structures for the FDTD simulations.

## Supplementary Material

Supplementary Material
