# Supplementary material for: Photonic crystal L3 cavity laser fabricated using maskless digital photolithography
Source: Nanophotonics. 2022 Apr 11;11(10):2283–91. doi: 10.1515/nanoph-2022-0021 (PMC11636002; doi:10.1515/nanoph-2022-0021)

[Supplementary Information]

Photonic crystal L3 cavity laser fabricated using maskless digital photolithography

Minsu Kang^1,2^, HeeSoo Jin^1,2^, and Heonsu Jeon^1,2,3^*

^1^Department of Physics and Astronomy, Seoul National University, Seoul 08826, Republic of Korea

^2^Inter-university Semiconductor Research Center, Seoul National University, Seoul 08826, Republic of Korea

^3^Institute of Applied Physics, Seoul National University, Seoul 08826, Republic of Korea

*e-mail: hsjeon@snu.ac.kr

List of Contents

[S1. MDPL versus EBL 3](#_Toc99286379)

[S2. Determination of proper exposure times 4](#_Toc99286380)

[S3. Exposure dosage amplitude versus DMD pixel configuration 6](#_Toc99286381)

[S4. Exposure time adjustment in accordance with *N*_nn_ 8](#_Toc99286382)

[S5. Abnormality in hole shape 10](#_Toc99286383)

S1. MDPL versus EBL

In the table below, the performance specifications (both merits and demerits) of our MDPL are compared with those of commercial MDPL and EBL, the latter being presently the most common and popular nanolithographic method. It should be reminded that we have no intention to compete with EBL in terms of the smallest pattern size that can be defined. Our aim is rather to demonstrate that our MDPL far outperforms EBL in other aspects. The most prominent advantage is the writing time (thus throughput), which is shorter by roughly two orders of magnitude (from ~1 min for EBL to ~1 sec for MDPL). Additional advantages include the simplicity in system configurations (thus low cost) due to no need for high vacuum chamber and electron beam generation/control unit. On the other hand, the smallest pattern sizes obtainable using our MDPL is > 200 nm, which is at least 5 times smaller than that of commercial MDPL systems (> 1 μm).

| **Table S1. Specifications of MDPL and EBL** | | | |  |
| --- | --- | --- | --- | --- |
| **Lithography**  **system** | **Smallest**  **pattern size** | **Writing field**  **(without stitching or scanning)** | **Writing time** | **Remarks** |
| Commercial MDPL  (DL-1000; Nano System Solutions) | > 1 μm | 1024 × 768 μm^2^ | ~1 sec per exposure | • The writing field is determined by the DMD and objective lens in the system.  • The writing time is independent of the exposure area. |
| **Our MDPL** | **< 200 nm** | 54.8 × 30.8 μm^2^ | **~1 sec** per exposure  (for a typical PhC pattern) |  |
| EBL  (JBX-6300FS; JEOL) | < 10 nm | ~100 × 100 μm^2^ | ~1 min  (for a typical PhC pattern) | • Requires high vacuum chamber and electron beam unit |

S2. Determination of proper exposure times

Here we explain the details of how to determine the proper exposure time for the *n* = 3 case as an example, where each air-hole pattern is defined by 5 (= 3 × 3 − 4) DMD pixels. The proper exposure times for other *n*’s can be deduced similarly. Shown in Fig. S1a are the calculated *S*_on_, *S*_off_, and *S*_tot_ for *n* = 1. Let us first assume that the proper exposure time for *n* = 1, *t*_0_, is already known. Then the PR threshold would intersect the total exposure dosage profile somewhere in the middle, resulting in a periodic PhC pattern after PR development. Now, let us deduce the exposure dosage profiles for *n* = 3 when the exposure time still remains at *t*_0_, which is valid for *n* = 1. The calculation results are summarized in Fig. S1b. Because the exposure time is unchanged while the number of the ‘on’ DMD pixels is increased by a factor of 5, the total exposure dosage profile is well above the PR threshold. This infers that the PR is to be completely washed out when developed. Therefore, the exposure time should be reduced such that the PR threshold lines up again in the middle of the total exposure dosage profile. In case of *n* = 3, such a desirable situation can be achieved when *m* = 11, *i.e.*, when the exposure time is *t_−_*_11_ = *t*_0_(1.1)^−11^ ≈ 0.35*t*_0_. The corresponding situation is depicted in Fig. S1c. The appropriateness of the exposure time *t_−_*_11_ for *n* = 3 has been confirmed experimentally. Figure S1d displays the SEM photos of the PR patterns after development when the exposure times are *t_−_*_10_, *t_−_*_11_, *t_−_*_12_, and *t_−_*_13_, which turn out to be overexposed, proper, acceptable, and underexposed, respectively.

.

Fig. S1


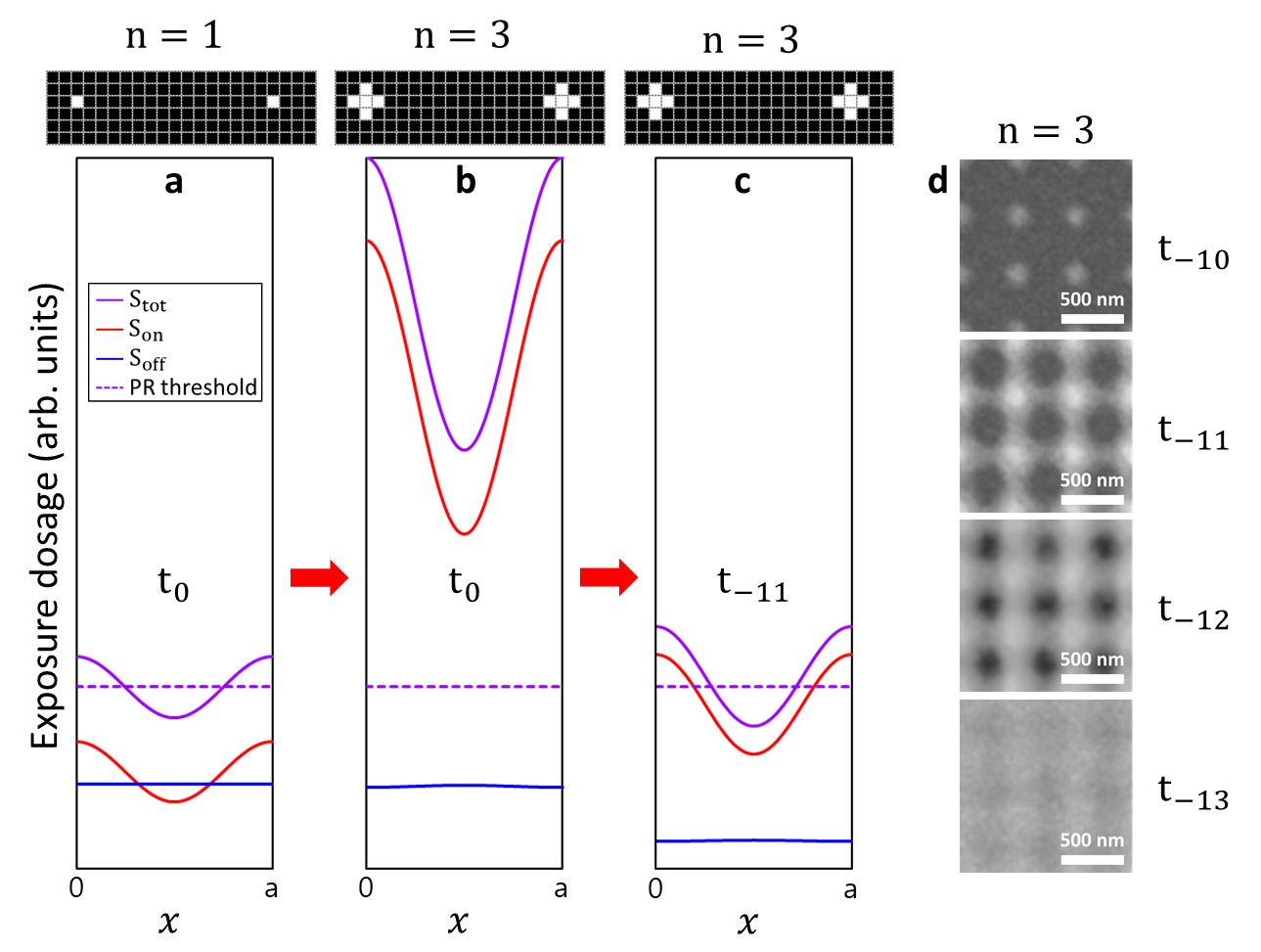


S3. Exposure dosage amplitude versus DMD pixel configuration

It is intuitively rather obvious that the FWHM of the exposure dosage profile for a single air-hole pattern increases monotonically as the number of the DMD pixels that define the air-hole pattern (or *n*) is increased; Fig. S2a shows the calculated FWHM as a function of *n*. Consequently, the overlap between adjacent air-hole patterns becomes progressively more significant as *n* gets larger. Figure S2b, which compares the exposure dosage profiles by the ‘on’ pixels for *n* = 1 and 6, confirms the expectation. The most noticeable change is that the valleys of the combined exposure dosage profile move up while the peaks remain practically unchanged, which effectively flattens out the overall exposure dosage profile and thus reduces the modulation amplitude Δ*S*_on_.

Fig. S2


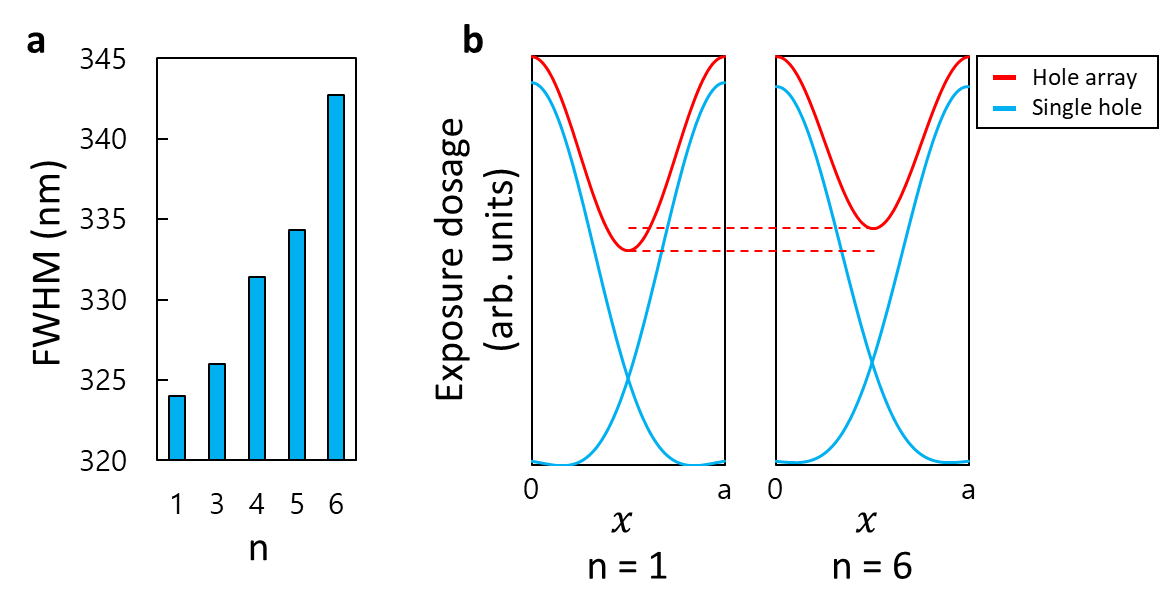


S4. Exposure time adjustment in accordance with *N*_nn_

As clarified in Fig. 3(e) and 3(f) of the main manuscript, light diffraction from the DMD pixels is significant enough to affect the formation of nearby air-hole patterns. Consequently, the size of an air-hole pattern heavily depends on the number of its nearest-neighbor air-holes, *N*_nn_. To make the air-hole patterns equal and uniform, therefore, it is necessary to adjust the exposure dosages for individual air-holes accordingly. Figure S3 shows how the air-hole pattern sizes (*r*/*a*) change for different *N*_nn_’s as a function of exposure dosage change (equivalently, exposure time change). Note that the air-hole pattern sizes at the 0% dosage adjustment correspond to the average values of those shown in Fig. 3(f) of the main manuscript. Our strategy is to increase the exposure dosage for *N*_nn_ = 3 and 4 and simultaneously to decrease it for *N*_nn_ = 5 and 6 so that all the air-hole pattern sizes line up at the target value, for example, *r*/*a* = 0.30. The appropriate exposure dosage adjustments turn out to be Δ*S* ≈ +7.59%, +2.74%, −2.56%, and −5.09% for *N*_nn_ = 3, 4, 5, and 6, respectively. The PR pattern after the exposure corrections is shown in Fig. 3(g) of the main manuscript, which is much improved in terms of the uniformity in air-hole sizes when compared with Fig. 3(e).

Fig. S3


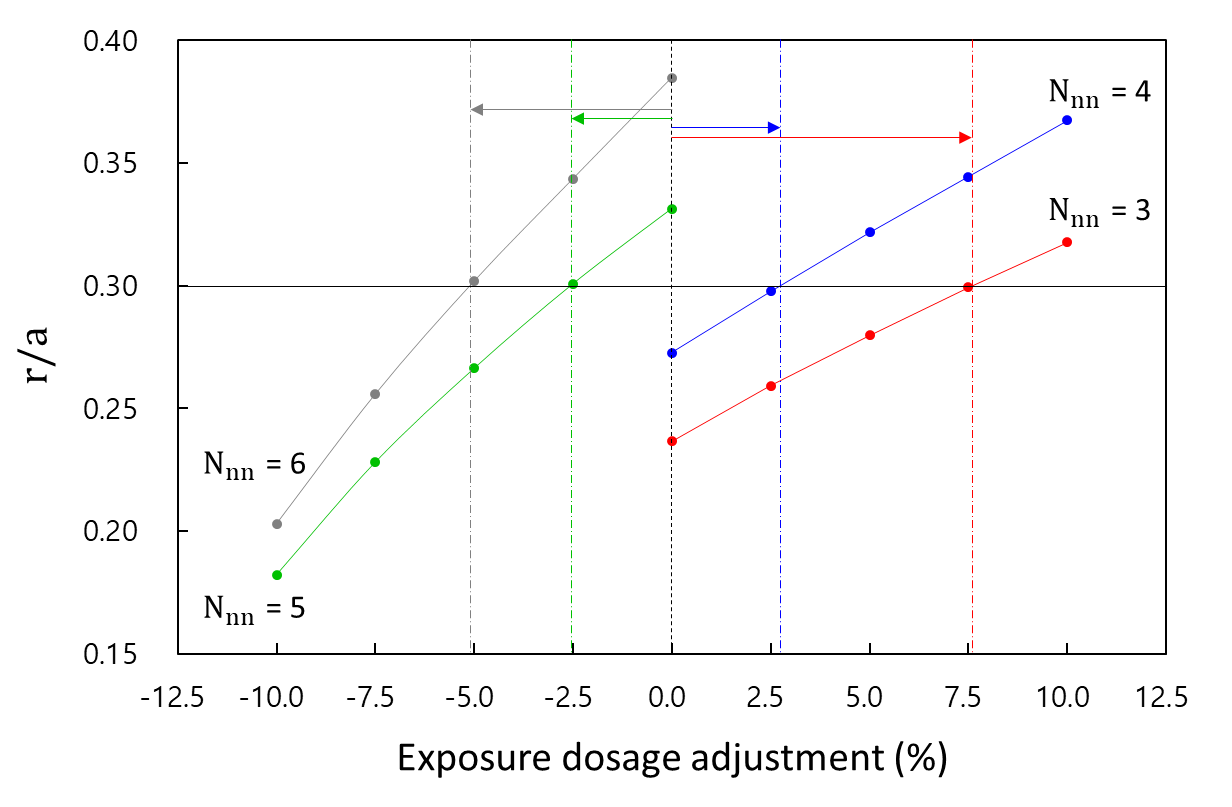


S5. Abnormality in hole shape

A close examination of Fig. 4a in the main manuscript reveals that the air holes (especially the smaller ones) are distorted in shape, which is quite important for the performance of resultant photonic devices. To quantify the abnormality of the air hole patterns, the SEM image in Fig. 4a is digitized (Fig. S4a) and compared with an artificial image composed of circular patterns only (Fig. S4b); the area of each hole pattern is kept the same. In Fig. S4c, the green area denotes the region where the SEM image protrudes out of the circle whereas the red area corresponds to the region dented inside. The abnormality factor in percentage is defined as $AN=\left[ {(A_{G}+A_{R})}/\left( A_{hole} \right) \right]\cdot100$, where *A_G_* and *A_R_* are the areas of the green and red regions, respectively. Figure S4d plots the abnormality factors obtained for the entire hole patterns. It is interesting to note that as the hole diameter becomes smaller, the abnormality factor increases (mainly due to diffraction). As already clarified in the main manuscript, the PhC patterns are improved by considering the nearest-neighbour effect, which is also true for the abnormality, as can be seen in Fig. S4e where the abnormality factor is plotted for all the hole patterns in Figs. 4a (before the correction) and 4b (after the correction) of the main manuscript. The average abnormality factors, denoted as the blue dots in the figures, are 5.63% and 4.35% before and after the nearest-neighbour correction, respectively. It is important to keep exerting this kind of efforts to improve the photolithographic roughness or irregularity.

Fig. S4


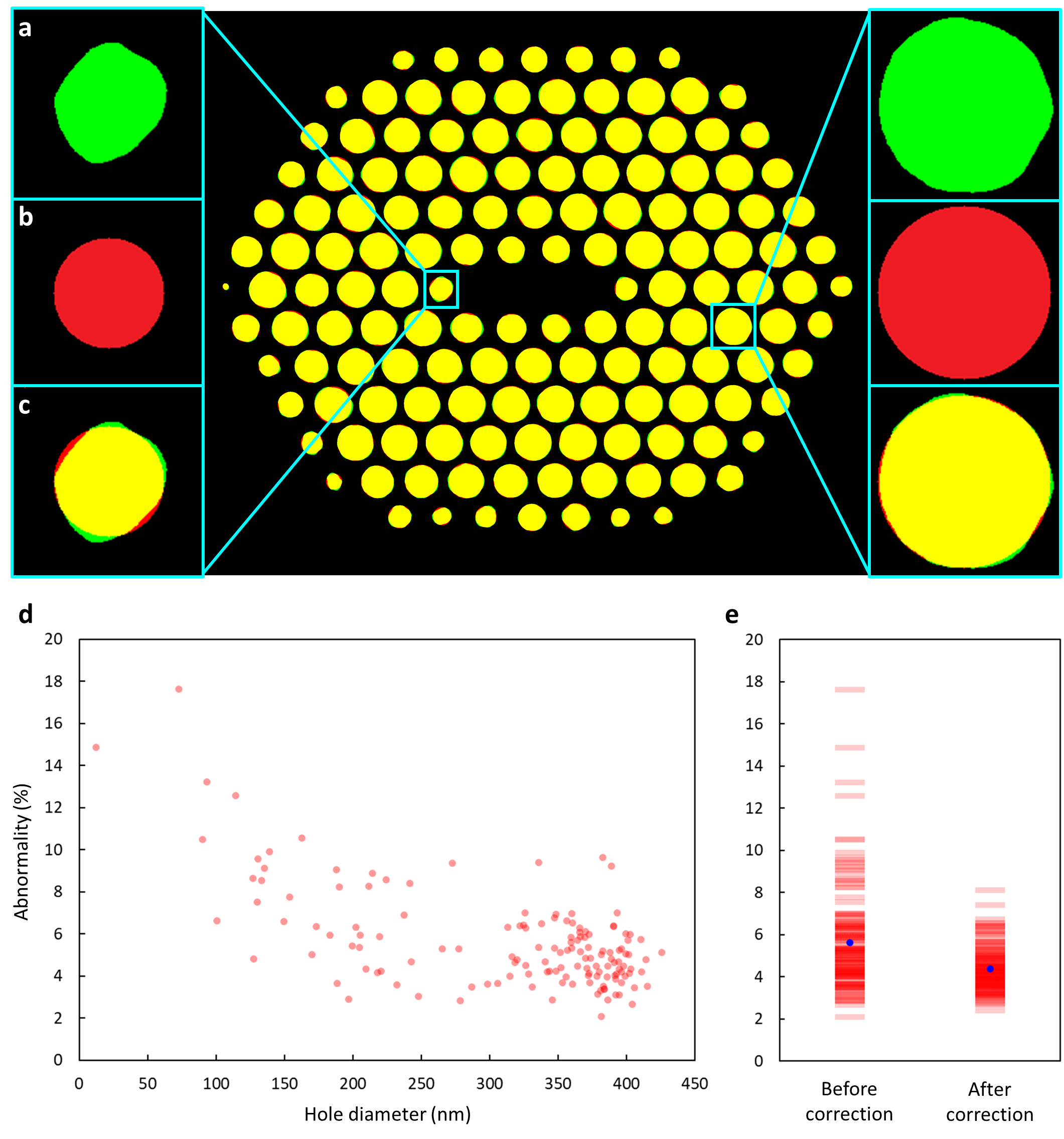

Supplement: Supplementary file 1 — Supplementary Material [file j_nanoph-2022-0021_suppl.docx]
